# Aspirin increases metabolism through germline signalling to extend the lifespan of *Caenorhabditis elegans*

**DOI:** 10.1371/journal.pone.0184027

**Published:** 2017-09-14

**Authors:** Xiao-Bing Huang, Xiao-Hui Mu, Qin-Li Wan, Xiao-Ming He, Gui-Sheng Wu, Huai-Rong Luo

**Affiliations:** 1 Key Laboratory for Aging and Regenerative Medicine, Department of Pharmacology, School of Pharmacy, Southwest Medical University, Luzhou, Sichuan, China; 2 State Key Laboratory of Phytochemistry and Plant Resources in West China, Kunming Institute of Botany, Chinese Academy of Sciences, Kunming, Yunnan, China; 3 Yunnan Key Laboratory of Natural Medicinal Chemistry, Kunming Institute of Botany, Chinese Academy of Sciences, Kunming, Yunnan, China; 4 University of Chinese Academy of Sciences, Beijing, China; East Carolina University, UNITED STATES

## Abstract

Aspirin is a prototypic cyclooxygenase inhibitor with a variety of beneficial effects on human health. It prevents age-related diseases and delays the aging process. Previous research has shown that aspirin might act through a dietary restriction-like mechanism to extend lifespan. To explore the mechanism of action of aspirin on aging, we determined the whole-genome expression profile of *Caenorhabditis elegans* treated with aspirin. Transcriptome analysis revealed the RNA levels of genes involved in metabolism were primarily increased. Reproduction has been reported to be associated with metabolism. We found that aspirin did not extend the lifespan or improve the heat stress resistance of germline mutants of *glp-1*. Furthermore, Oil Red O staining showed that aspirin treatment decreased lipid deposition and increased expression of lipid hydrolysis and fatty acid β-oxidation-related genes. The effect of germline ablation on lifespan was mainly mediated by DAF-12 and DAF-16. Next, we performed genetic analysis with a series of worm mutants and found that aspirin did not further extend the lifespans of *daf-12* and *daf-16* single mutants, *glp-1;daf-12* and *glp-1;daf-16* double mutants, or *glp-1;daf-12;daf-16* triple mutants. The results suggest that aspirin increase metabolism and regulate germline signalling to activate downstream DAF-12 and DAF-16 to extend lifespan.

## Introduction

Aspirin is a nonsteroidal anti-inflammatory medication used for the clinical treatment of multiple diseases, such as pain, fever, inflammation, platelet aggregation, and heart attacks, and in cancer prevention [1,2]. The long-term use of aspirin can ameliorate the onset of various age-related diseases and increase the maximum and mean lifespan in yeast, worms, mice, and humans [3–7]. Aspirin can inhibit oxidant production, cytokine responses, and glycation reactions and can suppress NF-κB to decrease inflammation and age-related reactive species [8–11]. In addition, aspirin can delay the onset of endothelial senescence by preventing decreases in NO generation [12]. However, the molecular mechanisms and the targets for the antiaging effect of aspirin remain obscure.

*Caenorhabditis elegans* (*C*. *elegans*) is an excellent model for studying the molecular mechanism of longevity modulation because of its short lifespan and amenability to genetic manipulation. Previous work has shown that aspirin attenuates the endogenous levels of reactive oxygen species and up-regulates the expression of antioxidant genes to reduce age-associated functional declines and extend the lifespan of *C*. *elegans* [3]. Further results showed that aspirin may act through a dietary restriction-like mechanism by activating AMPK, which is an energy sensor that can stimulate DAF-16 to induce downstream effects [7]. Recent results have shown that aspirin reduces SOD-1 aggregation in amyotrophic lateral sclerosis (ALS) and decreases amyloid aggregates in Alzheimer’s diseases (AD) by donating its acetyl to decrease phosphorylation, which suggests that acetylation might contribute to the neuroprotective and lifespan extension effects of aspirin [13]. Aspirin acts on the prostaglandin system via irreversible inhibition of cyclooxygenases to control pain, fever, and inflammation. Interestingly, there is no homologue of cyclooxygenases in *C*. *elegans*; thus, the mechanism of action of aspirin in *C*. *elegans* has yet to be revealed.

Here, we explore the mechanism of lifespan extension by aspirin in *C*. *elegans*. We found that aspirin regulated metabolic pathways to increase lipid hydrolysis, inhibited the proliferation of germline stem cells, and subsequently activated DAF-12 and DAF-16 to extend the lifespan of *C*. *elegans*.

## Results

### Aspirin up-regulates metabolic pathways

To reveal the effect of aspirin on *C*. *elegans*, we used RNA-Seq to analyse which processes were altered by aspirin in worms. Based on transcriptome data, we identified 77 differentially expressed genes within several genetic pathways involved in the aging process in worms treated with aspirin. Among these, 59 genes were significantly up-regulated, and 18 genes were down-regulated (S1A and S1B Fig). To confirm the results in RNA-Seq, we randomly chose 8 genes from 77 differentially expressed genes for qRT-PCR analysis. The transcriptional changes in these 8 genes as determined by qRT-PCR were consistent with our RNA-Seq results (S1C Fig).

**Fig 1:**
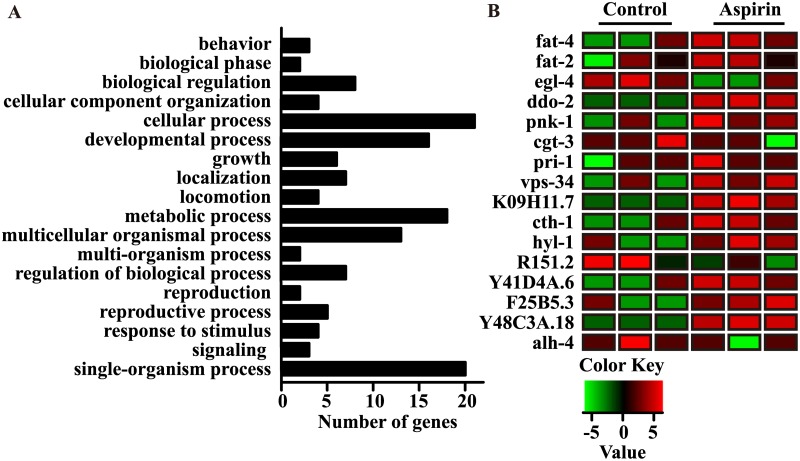
Aspirin regulates metabolic pathways. (A) GO analysis of differentially expressed genes. Differentially expressed genes were BLASTX against Gene Ontology to predict its function and were classified into GO functional groups by WEGO analysis. (B) Heat map of differentially expressed genes involved in metabolism in RNA-Seq of aspirin. Statistical details and repeats of these experiments are summarized in S1 Table.

GO analysis and classification showed that aspirin mainly affected cellular, developmental, metabolic, multicellular organismal, single-organismal and reproductive processes (Fig 1A). In our previous study, preliminary mechanistic investigation indicated that metabolic pathways might be involved in the regulation of aging by aspirin [3]. Accordingly, we further used KEGG analysis to identify the biological processes related to metabolism. KEGG analysis showed that several physiological processes involved in metabolism were dramatically changed, including metabolic pathways (*p* = 0.0278), purine metabolism (*p* = 0.0122), pyrimidine metabolism (*p* = 0.0101), aminoacyl-tRNA biosynthesis (*p* = 0.0350) and sphingolipid metabolism (*p* = 0.0365). The following metabolism-related genes were prominently up-regulated in worms treated with aspirin: the fatty acid desaturase genes *fat-2* and *fat-4*; genes involved in hydrolysis, such as *hyl-1*, *cth-1*, and K09H11.7; transportation genes, including *vps-34* and *cgt-3*; developmental genes, including *pri-1* and Y41D4A.6; and other biological process genes, including *ddo-2*, Y48C3A.18 and F25B5.3 (Fig 1B). We also found that the mRNA levels of several genes involved in lifespan regulation were significantly altered in aspirin-treated worms, such as the following: genes involved in the regulation of longevity and autophagy, including *pcm-1*; genes involved in development and reproduction, including *pnk-1*, *alh-4*, and *ccr-4*; and genes that are involved in many physiological processes, including *tiar-1*, *egl-4*, and *clpp-1*. In addition, some genes regulated by aspirin were involved in reproduction-related processes, including *rpt-4*, *vgln-1*, *xbx-6*, T08B2.5, Y17G7B.18, Y17G7B.20, and Y106G6H.6. Some of the genes with altered expressions were confirmed by qRT-PCR (S1C Fig). Because altered metabolic homeostasis is a hallmark of lifespan modulation [14], our transcriptome data suggested that aspirin may regulate metabolic pathways to exert its antiaging effect in *C*. *elegans*.

### Aspirin could not extend the lifespan of germline mutants

**Fig 2:**
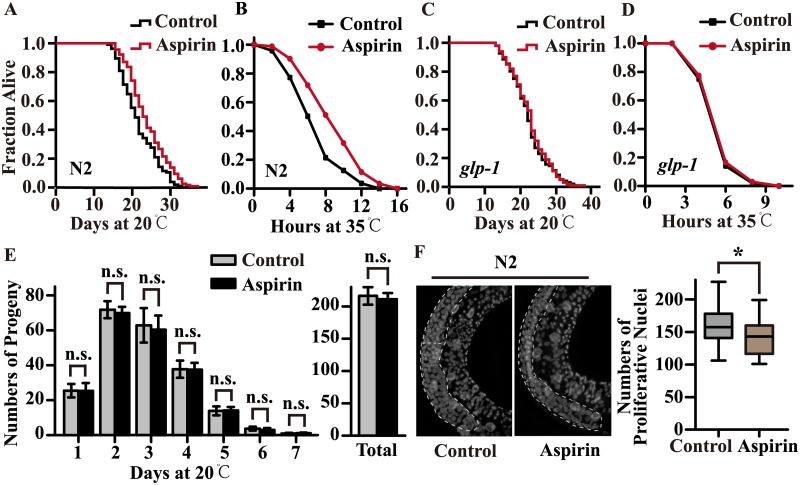
Aspirin does not extend the lifespan of germline mutants. The survival curves of (A) wild-type worms and (C) *glp-1(e2141)III*. raised at 20°C on NGM plates containing 100 μM aspirin or control. The survival curves of (B) wild-type worms and (D) *glp-1(e2141)III*. raised at 35°C on NGM plates containing either no aspirin or 100 μM aspirin in thermo-tolerance assays. The lifespan was analysed by the Kaplan-Meier test, and *p* values were calculated by the log-rank test. (E) The number of progeny each day and the total number of progeny of the worms treated with 100 μM aspirin. The figures show the mean value of three independent experiments, and the error bars represent the SEM. *P* values were calculated by a two-tailed t-test. Not significant was abbreviated as n.s., calculated by a two-tailed t-test. (F) The quantification of the proliferative nuclei of germline stem cells in wild-type N2 worms treated with 100 μM aspirin. *p* values were calculated by a two-tailed t-test, * *p* < 0.05. For box-and-whisker plots, the whiskers show the minima and the maxima within a 1.5 interquartile range (IQR). Statistical details and repeats of these experiments are summarized in S2, S3, S6 and S7 Tables.

Many genetic factors are involved in lifespan extension, such as dietary restriction (DR), decreased the activity of the insulin/insulin-like signalling (IIS) pathway and mitochondrial respiration [15]. Moreover, as ablation of germline precursor cells of the gonad is among the treatments known to have robust effects on lifespan extension [16,17]. Reproduction is an energetically costly process that has profound effects on metabolism [18]. These findings prompted us to explore whether germline signals might participate in lifespan extension by aspirin. Our previous work showed that aspirin could significantly extend the lifespan of wild type N2 worms (Fig 2A) and increase heat stress resistance (Fig 2B) [7]. When we examined the effect of aspirin on germline mutants of *glp-1(e2141)III*., our results showed that aspirin could not further extend the lifespan of *glp-1*,(Fig 2C) or improve its resistance to heat stress (Fig 2D).

Down-regulation of germline signalling might extend lifespan and decrease the production of progeny [19]. We found that aspirin could not decrease the daily progeny production or the total progeny of each worm (Fig 2E), though it did decrease the number of germline stem cells in worms (Fig 2F).

### Aspirin increased lipid hydrolysis and fatty acid β-oxidation in worms

**Fig 3:**
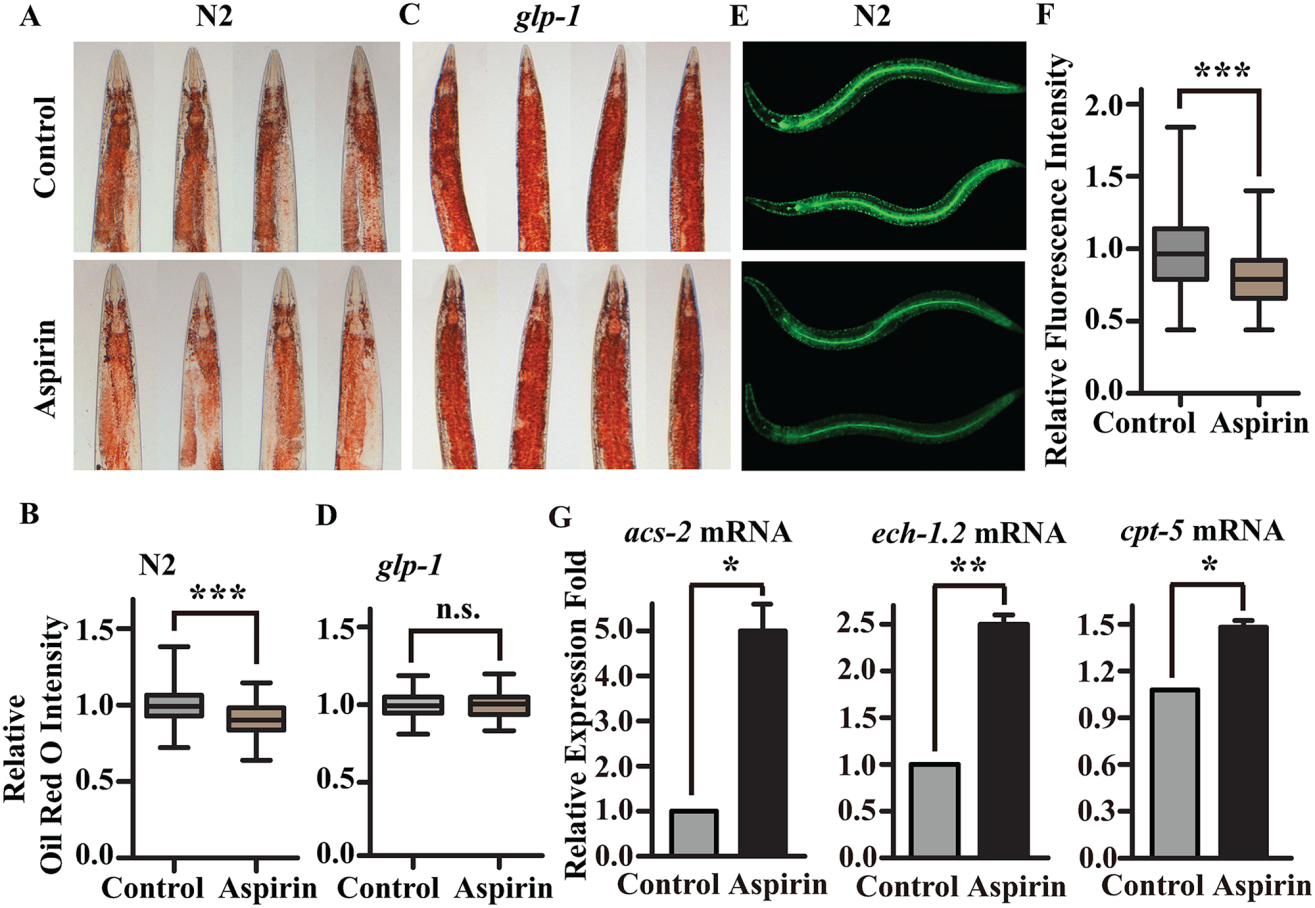
Aspirin increases lipid hydrolysis. Oil Red O Staining of (A) wild-type worms and (C) *glp-1(e2141)III*. mutants treated with 100 μM aspirin. The relative Oil Red O intensities of (B) wild-type worms and (D) *glp-1(e2141)III*. mutants were calculated using Image J. (E) Aspirin decreased the absorption of BODIPY- labelled fatty acids in wild-type N2 worms. (F) The relative GFP fluorescence intensity was calculated using Image J. For box-and-whisker plots, the whiskers show the minima and the maxima within a 1.5 IQR. (G) The mRNA level of the expression of enzymes in fatty acid β-oxidation of wild-type worms treated with or without 100 μM aspirin. The figures show the mean value of three independent experiments, and the error bars represent the SEM. *P* values were calculated by a two-tailed t-test, * *p* < 0.05, ** *p* < 0.01. Not significant was abbreviated as n.s. Statistical details and repeats of these experiments are summarized in S4, S5 and S8 Tables.

Germline removal in the nematode promotes longevity in part by modulating lipid metabolism through effects on fatty acid desaturation, lipolysis, and autophagy [18,20]. Results of transcriptome sequencing revealed that aspirin mainly regulated metabolic processes; moreover, aspirin could not extend the lifespan of germline mutants. Therefore, we examined whether aspirin influenced lipid metabolism and absorption in worms. Oil Red O staining showed that aspirin significantly decreased lipid disposition in wild-type N2 worms (Fig 3A and 3B), but not in the germline mutant *glp-1(e2141)III*. (Fig 3C and 3D). Aspirin also significantly decreased the absorption of BODIPY-labelled fatty acids (Fig 3E and 3F). To investigate whether aspirin increased lipid metabolism, we measured the expression of genes that encode the enzymes for fatty acid β-oxidation [21]. ACS-2 encodes acyl-CoA synthase and is responsible for catalysing the conversion of fatty acid to acyl-CoA for subsequent β-oxidation in worms [22]. Our results showed that aspirin increased *acs-2* expression by nearly five-fold (Fig 3G). Aspirin also increased the expression levels of *ech-1*.*2* and *cpt-5*, the genes that encode enoyl-CoA hydratase and carnitine palmitoyl transferase, respectively (Fig 3G), suggesting that aspirin increased lipid hydrolysis and reduced fat storage in *C*. *elegans*.

### Aspirin requires DAF-12 and DAF-16 to extend the lifespan of *C*. *elegans*

**Fig 4:**
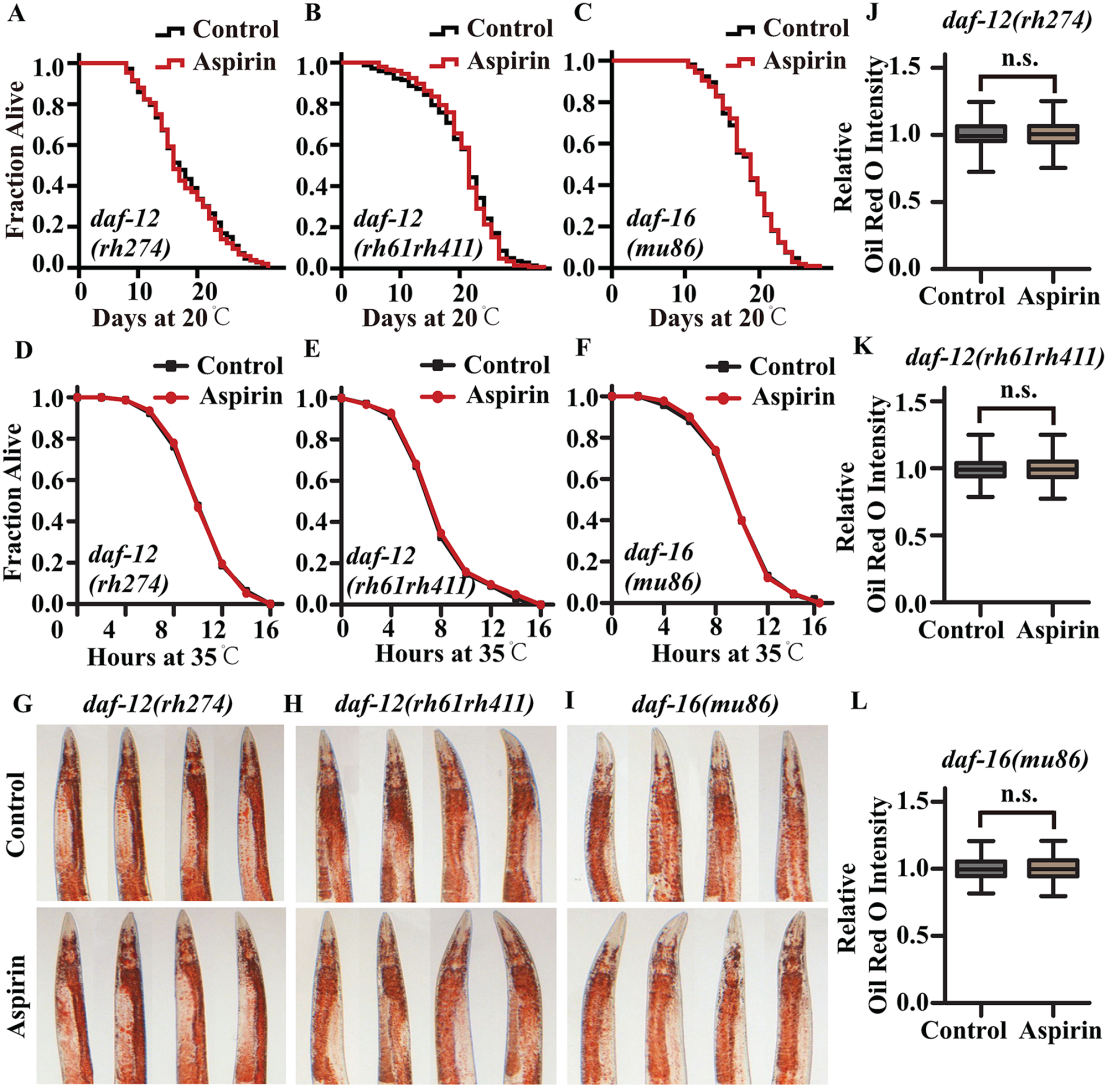
Aspirin increases lipid hydrolysis and extends the lifespan of *C*. *elegans*, requiring DAF-12 and DAF-16. Survival curves of (A) *daf-12(rh274)X*., (B) *daf-12(rh61rh411)X*., and (C) *daf-16(mu86)I*. cultured at 20°C on NGM plates containing either no aspirin or 100 μM aspirin in lifespan assays. Survival curves of (D) *daf-12(rh274)X*., (E) *daf-12(rh61rh411)X*., and (F) *daf-16(mu86)I*. raised at 35°C on NGM plates containing either no aspirin or 100 μM aspirin in thermo-tolerance assays. The lifespan was analysed by the Kaplan-Meier test, and *p* values were calculated by the log-rank test. Oil Red O Staining of (G) *daf-12(rh274)X*., (H) *daf-12(rh61rh411)X*. and (I) *daf-16(mu86)I*. mutants treated with 100 μM aspirin. The relative Oil Red O intensities of (J) *daf-12(rh274)X*., (K) *daf-12(rh61rh411)X*. and (L) *daf-16(mu86)I*. mutants were calculated using Image J. n.s., not significant with a two-tailed t-test. For box-and-whisker plots, the whiskers show the minima and maxima within a 1.5 IQR. Statistical details and repeats of the experiments are summarized in S2, S3 and S4 Tables.

DAF-12 is a nuclear hormone receptor and plays an important role in metabolism, developmental timing, longevity and the choice between reproductive development and arrest in worms [23]. DAF-16 is the mammalian homologue of the FOXO transcription factor and plays a central role in the mediation of development, reproduction, fat storage, stress resistance, and longevity [24]. The effect of germline ablation on lifespan extension was mainly mediated by DAF-12 and DAF-16 [16,25]. We questioned whether DAF-12 and DAF-16 play a role in the lipid hydrolysis and the lifespan extension mediated by aspirin. We found that aspirin could not extend the lifespan of two *daf-12* mutants, *daf-12(rh274)X*. (Fig 4A) and *daf-12(rh61rh411)X*. (Fig 4B), or that of the *daf-16* mutant *daf-16(mu86)I*. (Fig 4C). In addition, aspirin could not improve heat stress resistance in *daf-12* or *daf-16* mutants (Fig 4D–4F). Because DAF-12 and DAF-16 activate fat-processing enzymes to increase lipid hydrolysis in response to germline ablation in worms [26–28], we analysed the fat content of mutants when treated with aspirin. Our results showed that aspirin could not alter the fat content in *daf-12* mutants (Fig 4G–4K) or in *daf-16* mutants (Fig 4I–4L).

**Fig 5:**
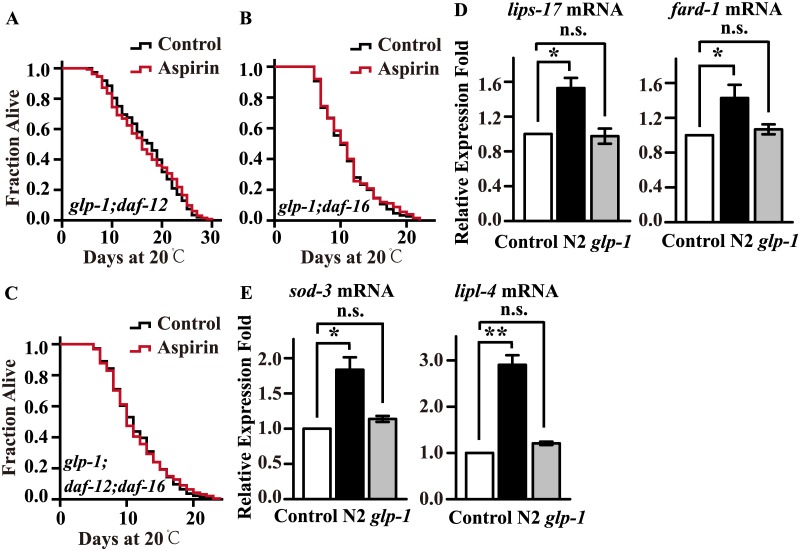
Aspirin regulates germline signalling to activate downstream DAF-12 and DAF-16. Survival curves of (A) *glp-1(e2141)III*.; *daf-12(rh61rh411)X*., (B) *glp-1(e2141)III*.; *daf-16(mu86)I*., and (C) *glp-1(e2141)III*.; *daf-12(rh61rh411)X*.; *daf-16(mu86)I*. raised at 20°C on NGM plates containing 100 μM aspirin or control. Lifespan was analysed using the Kaplan-Meier test, and *p* values were calculated using the log-rank test. The mRNA levels of (D) DAF-12- and (E) DAF-16-targeted genes in wild-type N2 worms and the mutant *glp-1(e2141)III*. treated with or without 100 μM aspirin. The figures show the mean value of at least three independent experiments and the error bars represent the SEM. *p* values were calculated by a two-tailed t-test, * *p* < 0.05, ** *p* < 0.01, not significant was abbreviated as n.s. Statistical details and repeats of these experiments are summarized in S2 and S8 Tables.

To investigate whether the signals from the reproductive system regulated the lipid metabolism and lifespan extension via DAF-12 and DAF-16, we constructed double and triple mutants of worms with germline mutants of *glp-1* and mutants of *daf-12* and *daf-16*. Our results showed that aspirin could not further extend the lifespan of the double mutants *glp-1;daf-12* (Fig 5A) or *glp-1;daf-16* (Fig 5B) or that of the triple mutant *glp-1;daf-12;daf-16* (Fig 5C). We compared our differentially expressed genes data with that from previous studies of DA-16 and DAF-12 [23,24]; and found target genes with altered expression, including *brp-1*, R151.2, *lit-1*, *fat-2*, *ctns-1*, *egl-4*, *vgln-1*, Y73B6BL.31, *pnk-1*, R31.2, F27D4.4, *rpl-22*, and F41D9.2. Furthermore, we measured and confirmed the expression levels of DAF-12- and DAF-16-regulated genes: *lips-17* and *fard-1* for DAF-12 and *sod-3* and *lipl-4* for DAF-16 by qRT-PCR [29]. Our results showed that aspirin treatment significantly increased the mRNA levels of these genes in wild-type N2 worms but not in *glp-1* mutants (Fig 5D and 5E). The above results suggest that aspirin regulate germline signalling and activate its downstream transcription factors DAF-12 and DAF-16 to increase lipid metabolism and extend the lifespan of *C*. *elegans*.

## Discussion

Recent studies have shown that aspirin can delay the aging process and increase the lifespans of mice and *C*. *elegans* [3,6,7]. To explore the mechanism of aspirin on aging, we determined the whole-genome expression profile of *C*. *elegans* treated with aspirin. Transcriptome analysis revealed that the RNA levels of genes involved in metabolism increased. Aspirin may increase lipid hydrolysis by up-regulating the genes of fatty acid degradation to preserve a low energy status and extend the lifespan and improve the health of worms.

Research has shown that the ablation of germ cell progenitors, which are the source of adult germ cells in the gonad, remarkably prolongs longevity, suggesting that a signal might be released from germ cells that normally accelerate senescence and that the loss of this signal may extend lifespan [25]. We questioned whether aspirin could inhibit the activity of germline stem cells. Our results showed that aspirin reduced the number of proliferative zone nuclei, but the number of offspring remained unaltered. One possible explanation is that the successful hatch rate might be slightly higher in worms treated with aspirin than in controls. The reduced number of proliferative zone nuclei might decrease the signalling that accelerates aging processes in worms [16]. Therefore, decreases in germline stem cells might contribute aspirin’s ability to retard senescence. Nonetheless, more works should be carried out to confirm this hypothesis. The effect of germline ablation on longevity principally depends on the presence of the transcription factors DAF-12 and DAF-16. Aspirin increased the expression levels of the DAF-12-regulated gene *lips-17* (encoding hydrolase) and the DAF-16-regulated gene *lipl-4*, which encodes a triglyceride lipase and plays a role in lipid hydrolysis [29], suggesting that aspirin increased lipid hydrolysis mainly through DAF-12 and DAF-16. These results suggest that aspirin has effects on reproduction-related physiology.

Salicylate and acetylsalicylic acid are reported to initially exert their therapeutic effects through inhibition of cyclooxygenases and modulation of NF-κB activity [30–32]. Recent studies have shown that salicylate can directly activate AMPK [33] and can uncouple mitochondria, improve glucose homeostasis, and reduce liver lipids independent of AMPK [34]. The primary metabolite of aspirin (acetyl SA) SA can bind to plant and human glyceraldehyde 3-phosphate dehydrogenase (GAPDH), which plays a central role in glycolysis and suppresses nuclear translocation [35]. Similar results showed that aspirin could acetylate and inhibit glucose-6-phosphate dehydrogenase (G6PD), which catalyses the first reaction in the pentose phosphate pathway and is important for the regulation of oxidative stress [36]. For the mechanism of action in anti-cancer and chemoprevention activities, aspirin and salicylic acid can directly bind to CDK2 and down-regulate cyclin A2/CDK2 proteins [37] and inhibit CBP and p300 lysine acetyltransferase activity in vitro through direct competition with acetyl-Coenzyme A at the catalytic site [38]. These results revealed that the targets of aspirin and its metabolite are associated with metabolic function or immunity and are therefore involved in aging modulation. Our previous study and our current results show that multiple pathways are involved in the regulation of the aging process by aspirin. Our study also shows that pathway analysis may be limited in its ability to reveal the precise mechanism of aspirin, given the multiple potential action sites of aspirin. Our results indicate that aspirin increases lipid metabolism through germline signalling to activate downstream transcription factors DAF-12 and DAF-16 to extend the lifespan of *C*. *elegans*. Aspirin has been widely used for the prevention of deterioration in age-related disease. Therefore, further investigations are warranted on the mechanism by which aspirin influences the aging process and its clinical application. This work may provide clues for further discoveries on the function of aspirin in aging.

## Materials and methods

### Chemical and strains

The strains were obtained from Caenorhabditis Genetics Center (CGC) and maintained at an appropriate temperature. The strains used in this study were as follows: N2 (Bristol, wild type), CF1903 *glp-1(e2141)III*., CF1038 *daf-16(mu86)I*., AA86 *daf-12(rh61rh411)X*., AA89 *daf-12(rh274)X*., CF1880 *glp-1(e2141)III*.; *daf-16(mu86)I*., CF2248 *glp-1(e2141)III*.; *daf-12(rh61rh411)X*.; *daf-16(mu86)I*.; *muEx158*., *glp-1(e2141)III*.;*daf-12(rh61rh411)X*. All strains were maintained and grown on NGM plates seeded with *Escherichia coli* OP50.

Aspirin was purchased from Sigma and dissolved in PBS. NGM plates containing aspirin were equilibrated overnight before use.

### Lifespan assay

All strains were cultured on fresh NGM plates for 2–3 generations without starvation, and lifespan analysis was conducted at 20°C, unless otherwise stated. Late L4 larvae or young adults were transferred to NGM plates containing inactivated OP50 (65°C for 30 min) and 20 μM FUdR to inhibit progeny growth [7,39]. The day that L4 larvae or young adults were transferred to an NGM plate was defined as test day 0. Then, the worms were transferred to fresh plates every other day. For the strains of CF1903, CF1880, CF2248 and *glp-1(e2141)III*.;*daf-12(rh61rh411)X*., L1 worms were cultured at 20°C for 6 h, switched to 25°C until they developed into L4 larvae or young adults, and then switched back to 20°C for the lifespan test [40]. The worms that did not respond to a mechanical stimulus were scored as dead. The worms were censored if they crawled off the plate, displayed extruded internal organs, or died from hatching progeny inside the uterus. All lifespan assays were repeated in at least three independent trials.

### Transcriptome sequencing

Total RNA was extracted using RNAiso Plus (Takara) and treated with RNAase-free DNase I. The mRNA was isolated and enriched using oligo (dT) magnetic beads. First-strand cDNA was generated using random hexamer-primed reverse transcription, followed by synthesis of the second-strand cDNA using RNase H and DNA polymerase I. Then, single-end and paired-end RNA-Seq libraries were prepared following Illumina’s protocols and sequenced on the Illumina HiSeq^™^ 2000.

For quality control, raw reads were processed to obtain high-quality, clean reads by removing adaptor sequences, ambiguous reads, and low-quality reads (more than half of the base quality less than 5). After data quality assessment, clean reads were mapped to reference sequences using BWA and Bowtie. The average alignment rate was 86.16%. To eliminate the influence of different gene lengths and sequencing discrepancies on the calculation of gene expression, the FPKM (fragments per kilobase of transcript per million fragments mapped) method was used to valuate gene expression levels [41]. Genes with a FPKM ratio of the two samples above 2 and a Benjamini FDR (False Discovery Rate) ≤0.001 were defined as differentially expressed genes between the control group and aspirin-treated group.

Differentially expressed genes were assigned into gene groups and functional categories using Database for Annotation, Visualization and Integrated Discovery (DAVID), Gene Ontology (GO) and the Kyoto Encyclopedia of Genes and Genomes (KEGG) enrichment analyses [42,43].Raw data files are reserved in the NCBI Sequence Read Archive (SRP081065).

### Oil Red O staining

Oil Red O staining was conducted according to a previous report [44]. Briefly, adult worms were collected by washing the plates with PBS and were cleaned by further washing with PBS. Then, the worms were incubated with MRWB buffer containing 1% paraformaldehyde (PFA) at room temperature for 1 h. Next, the worms were dehydrated by incubation with 60% isopropanol for 15 min. Subsequently, the worms were stained by incubation with 60% Oil Red O stain for 2 h. Finally, the animals were mounted and imaged with a microscope system (Olympus, IX51). The images were analysed in a green channel using Image J software as previously reported [45]. More than 40 worms were measured for each experiment, and the experiments were repeated at least 3 times.

### BODIPY C12 uptake assay

Day-one adults were placed on 3.5-cm NGM plates containing 100 μl of 5 μM C1-BODIPY-C12 (Invitrogen) for 1 h at 20°C and then collected and washed with M9 three times [46]. The worms were mounted and imaged with a fluorescence microscope system (Olympus, IX51). Fluorescence intensity was measured using Image J.

### Thermos-tolerance assay

For the thermos-tolerance assay, L4 larvae or young adults were transferred to NGM plates with or without 100 μM aspirin. Then, the worms were transferred to fresh plates every day. On day five, the worms were incubated at 35°C and monitored for survival [39,47]. The animals were scored as dead when they did not respond to a gentle touch with a platinum wire pick every 2 h. At least 50 worms were used for each experiment. All thermos-tolerance assays were repeated in at least three independent trials.

### Reproduction assay

Synchronized L4 larvae or young adult worms were transferred to NGM plates containing 100 μM aspirin. Each plate contained one worm that was transferred to a fresh NGM plate at the same time each day. The offspring yielded by each worm on each day were hatched at 25°C and counted at the L4 stage [40]. The number of N2 worms used in each experiment was more than 20, and the experiments were repeated three times.

### Germline stem cell counting

Synchronized L4 larvae or young adult worms were washed from the bacterial lawn of plates with M9 buffer and stained using 4,6-diamidino-2-phenylindole (DAPI) as previously described [48,49]. Briefly, the worms were washed in M9 and fixed for 10 min in methanol at -20°C and then washed in M9 and stained with DAPI for 30 min. Images were acquired with a microscope system (Olympus, IX51). The numbers of proliferative zone nuclei included all germ cell nuclei between the distal tip and the beginning of meiotic entry, defined as the first row of cells in which two or more nuclei displayed the characteristic crescent shape as previously described [49]. At least 30 worms were used for each experiment, and the experiments were repeated three times.

### Gene expression assay

Approximately 2,000 synchronized young adult worms were transferred to NGM plates (9-cm diameter), with or without 100 μM aspirin, and cultured at 20°C for 24 h. Total RNA was extracted using RNAiso Plus (Takara) and converted to cDNA using a High Capacity cDNA Reverse Transcription Kit (Applied Biosystems). The qRT-PCR reactions were performed using Power SYBR Green PCR Master Mix (Applied Biosystems) and the ABI 7500 system. The relative expression levels of the genes were evaluated using the 2^–ΔΔCT^ method and normalized to the expression of the *cdc-42* gene [50]. The primers used here are listed in S9 Table.

### Statistical analyses

Lifespan statistical analyses were performed using the SPSS package. Kaplan-Meier lifespan analysis was performed, and *p* values were calculated using the log-rank test. Other results are expressed as the mean ± SEM and *p* values were calculated by a two-tailed t-test. *p* < 0.05 was considered significant.

## Supporting information

S1 Fig RNA-Seq results in wild-type worms treated with aspirin. (A) Differentially expressed genes in wild-type worms treated with aspirin. (B) Heat map of all differentially expressed genes in the RNA-Seq results of aspirin. (C) Differentially expressed genes and mRNA levels in wild-type worms treated with aspirin in RNA-Seq and qRT-PCR.
(PDF)

S1 Table The effects of aspirin on differentially expressed genes. (PDF)

S2 Table The effects of aspirin on lifespan. (PDF)

S3 Table The effects of aspirin on heat resistance. (PDF)

S4 Table The effects of aspirin on lipid metabolism. (PDF)

S5 Table The effects of aspirin on absorption. (PDF)

S6 Table The effects of aspirin on reproduction. (PDF)

S7 Table The effects of aspirin on the number of proliferative nuclei. (PDF)

S8 Table The effects of aspirin on mRNA expression. (PDF)

S9 Table Primer sequences of the genes used in the experiment. (PDF)
