# Supplementary figures and images for: Aspirin increases metabolism through germline signalling to extend the lifespan of Caenorhabditis elegans
Source: PLoS One. 2017 Sep 14;12(9):e0184027. doi: 10.1371/journal.pone.0184027 (PMC5598954; doi:10.1371/journal.pone.0184027)

Supplementary Figure 1

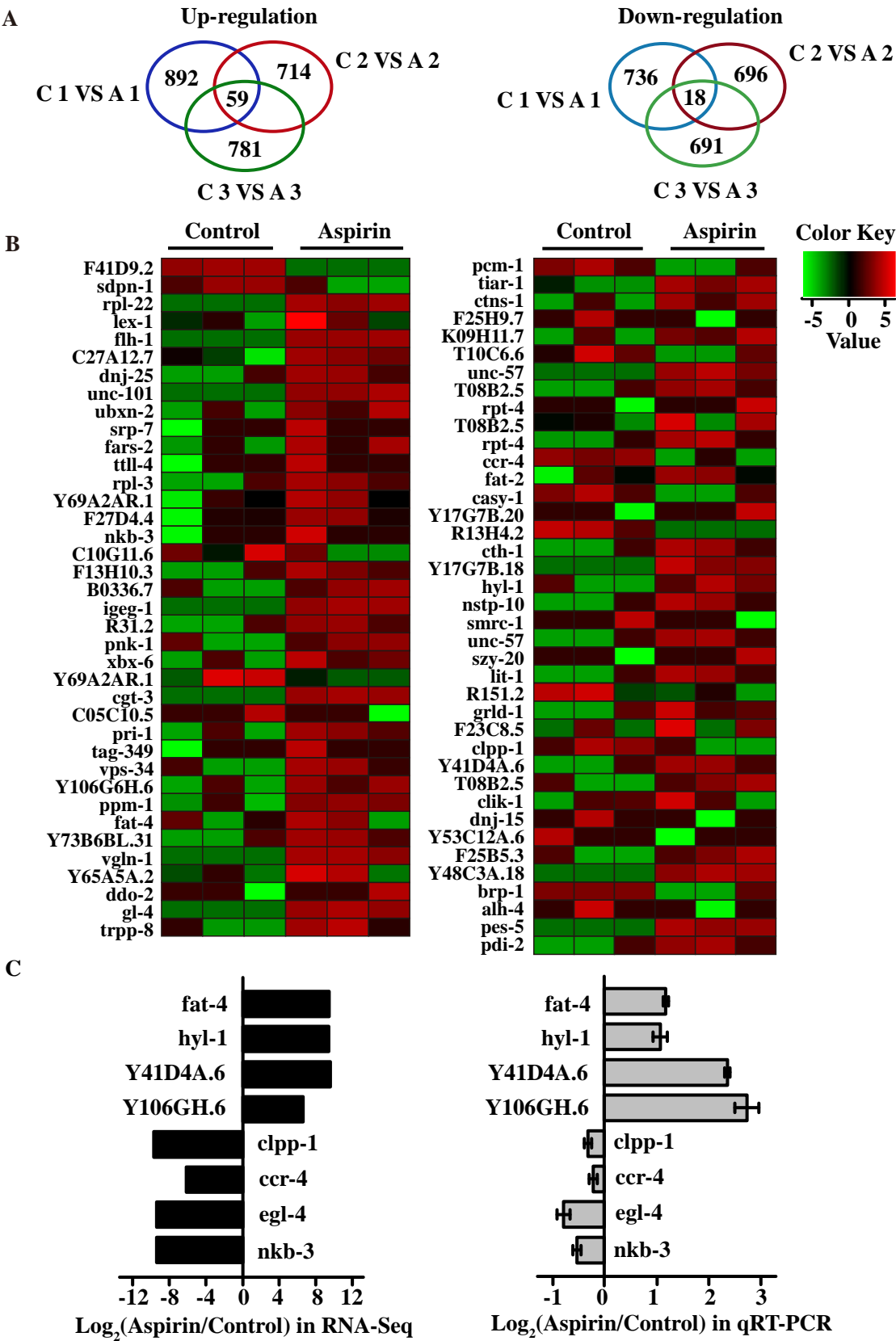

Supplement: S1 Fig — (A) Differentially expressed genes in wild-type worms treated with aspirin. (B) Heat map of all differentially expressed genes in the RNA-Seq results of aspirin. (C) Differentially expressed genes and mRNA levels in wild-type worms treated with aspirin in RNA-Seq and qRT-PCR. (PDF) [file pone.0184027.s001.pdf]
