# Supplementary material for: Aspirin increases metabolism through germline signalling to extend the lifespan of Caenorhabditis elegans
Source: PLoS One. 2017 Sep 14;12(9):e0184027. doi: 10.1371/journal.pone.0184027 (PMC5598954; doi:10.1371/journal.pone.0184027)
Supplement: S1 Table — (PDF) [file pone.0184027.s002.pdf]

Supplementary Table 1

Transcript	WormBase Gene ID	Description	Log_2 Ratio (Aspirin/Control)	Status
pdi-2	WBGene00003963	Oxidative protein folding in the endoplasmic reticulum	9.9019	Up
pes-5	WBGene00003979	Transposon	9.3000	Up
alh-4	WBGene00000110	Determination of adult lifespan, embryo development, nematode larval development and reproduction	-10.1699	Down
brp-1	WBGene00000273	Activate the mating pheromone responsive genes	-3.3008	Down
Y48C3A.18	WBGene00012997	Double-stranded RNA binding activity	9.5875	Up
F25B5.3	WBGene00017775	Orthologous to the human gene NT5C3	9.2432	Up
Y53C12A.6	WBGene00013141	Unclarified	-9.3007	Down
dnj-15	WBGene00001033	Encodes a protein containing a DnaJ ('J') domain	-9.2487	Down
clik-1	WBGene00020808	Orthologous to the human gene CNN and TAGLN	5.4686	Up
T08B2.5	WBGene00020346	Reproduction	9.1557	Up
Y41D4A.6	WBGene00021508	Development, lipid storage, and carbon-nitrogen ligase activity	9.5629	Up
clpp-1	WBGene00014172	Encodes a mitochondrial protein	-9.7296	Down
F23C8.5	WBGene00017734	Orthologous to the human gene ETFB	5.8074	Up
grld-1	WBGene00017929	Orthologous to the human gene RBM15	9.7392	Up
R151.2	WBGene00020107	Orthologous to the human gene PRPS1	-8.6264	Down
lit-1	WBGene00003048	Controlling the asymmetry of cell divisions during embryogenesis	10.4023	Up
szy-20	WBGene00004105	Negatively regulate centrosome size,	9.2946	Up

		duplication, and microtubule-nucleating capacity		
smrc-1	WBGene00015806	Remodeling of chromatin	-9.4485	Down
nstp-10	WBGene00008237	Orthologous to the human gene LAD II	11.0562	Up
hyl-1	WBGene00002043	Regulate lipid, particularly ceramide biosynthesis, regulate of lipid transport, and protein translocation in the endoplasmic reticulum	9.3627	Up
Y17G7B.18	WBGene00012469	Methyltransferase activity	9.9371	Up
cth-1	WBGene00009048	Encodes a putative cystathionine gamma-lyase	10.5038	Up
R13H4.2	WBGene00014826	Non-coding transcript isoform	-10.5850	Down
Y17G7B.20	WBGene00012471	Body morphogenesis, positive regulation of growth and reproduction	10.0357	Up
casy-1	WBGene00000403	Learning, embryonic development, and proper chemotactic behavior	-9.7027	Down
fat-2	WBGene00001394	Encodes a delta-12 fatty acyl desaturase	3.4562	Up
ccr-4	WBGene00000376	Development, determination of adult lifespan, embryo development, locomotion, positive regulation of growth and reproduction	-6.1817	Down
rpt-4	WBGene00004504	Encodes a predicted ATPase subunit of the 19S regulatory complex of the proteasome	12.6038	Up
unc-57	WBGene00006791	Synaptic vesicle endocytosis	10.3197	Up
T10C6.6	WBGene00011688	Transporter activity	9.6141	Up
K09H11.7	WBGene00019604	Phosphatase activity	9.4831	Up
F25H9.7	WBGene00009139	Orthologous to the human gene ACN9	-9.5085	Down
ctns-1	WBGene00008052	Lysosomal cystine transporter	9.3212	Up

tiar-1	WBGene00015943	Body morphogenesis, determination of adult lifespan, and lots of physiological process	-9.2846	Down
pcm-1	WBGene00003954	Longevity and autophagy	10.1745	Up
trpp-8	WBGene00018512	Intracellular transport and meiosis	9.2881	Up
egl-4	WBGene00001173	Encodes a cyclic GMP-dependent protein kinase	-9.4512	Down
Y106G6H.6	WBGene00013719	Embryo development and reproduction	6.5433	Up
ddo-2	WBGene00017565	Highest catalytic efficiency on D-aspartate or D-glutamate	9.6287	Up
Y65A5A.2	WBGene00013419	Unclarified	9.1699	Up
vgln-1	WBGene00007463	Body morphogenesis, embryo development and locomotion	3.4898	Up
Y73B6BL.31	WBGene00022252	Orthologous to the human gene SLC35	11.0240	Up
fat-4	WBGene00001396	Encodes a delta-5 fatty acid desaturase	9.4214	Up
ppm-1	WBGene00006460	Serine/threonine phosphatase activity	4.0814	Up
vps-34	WBGene00006932	Vesicular trafficking	9.3106	Up
tag-349	WBGene00013538	Ubiquitin-protein transferase activity	5.2524	Up
pri-1	WBGene00004180	Embryos for the normal timing of embryonic cell divisions	9.4023	Up
C05C10.5	WBGene00007332	Normal body morphology, chromosome segregation, and embryonic development	5.0634	Up
cgt-3	WBGene00019127	Synthesize glucosylceramide	-9.1948	Down
Y69A2AR.1	WBGene00022074	Unclarified	10.3436	Up
xbx-6	WBGene00009580	Orthologous to the human gene FAIM2, TMBIM1 and GRINA	-12.3817	Down
pnk-1	WBGene00004068	Apoptotic process, determination of adult lifespan, embryo development,	10.0444	Up

		lipid storage, nematode larval development and reproduction		
R31.2	WBGene00011270	Localized to the striated muscle myosin thick filament	3.3783	Up
igeg-1	WBGene00017901	Unclarified	9.0989	Up
B0336.7	WBGene00015147	Encodes two isoforms of a protein with a THAP or THAP-like domain	9.7016	Up
F13H10.3	WBGene00008774	Orthologous to the human gene SLC38A9	9.4965	Up
C10G11.6	WBGene00015686	Unclarified	9.4066	Up
nkb-3	WBGene00010117	Orthologous to the human gene P-type ATPases family	-9.4228	Down
F27D4.4	WBGene00009189	Metal ion binding activity	10.6567	Up
rpl-3	WBGene00004414	Required for embryonic and larval viability, fertility, and general health	5.7253	Up
ttll-4	WBGene00014232	Initiate glutamyl chains	9.3249	Up
fars-2	WBGene00013361	ATP, magnesium ion, tRNA, and phenylalanine-tRNA ligase binding activity	9.3302	Up
srp-7	WBGene00005648	Endoplasmic reticulum unfolded protein response	11.8957	Up
ubxn-2	WBGene00022381	Encodes one of six *C. elegans* UBX domain-containing proteins	9.3287	Up
unc-101	WBGene00006829	Motility, egg-laying, negative regulation of LET-23 / EGF-receptor signaling	10.2212	Up
dnj-25	WBGene00001043	Clathrin-mediated endocytosis and for normal development	10.4942	Up
C27A12.7	WBGene00016157	Ubiquitin-protein transferase activity	9.6540	Up
flh-1	WBGene00012435	Body morphogenesis, embryo	6.6614	Up

		development, negative regulation of transcription		
lex-1	WBGene00008682	Encodes a protein that contains an ATPase domain and a bromo domain	8.1960	Up
rpl-22	WBGene00004434	Protein biosynthesis	10.2046	Up
sdpn-1	WBGene00018467	Localizes to basolateral recycling endosomes	-9.3376	Down
F41D9.2	WBGene00018282	Unclarified	-9.6789	Down
