# Supplementary material for: Aspirin increases metabolism through germline signalling to extend the lifespan of Caenorhabditis elegans
Source: PLoS One. 2017 Sep 14;12(9):e0184027. doi: 10.1371/journal.pone.0184027 (PMC5598954; doi:10.1371/journal.pone.0184027)
Supplement: S2 Table — (PDF) [file pone.0184027.s003.pdf]

Supplementary Table 2

Figure	Strains	Treatments	Mean Lifespan ± SEM (days)	P value VS Control	% Change in Mean Lifespan	N
	**N2 (WT)**					
**2(A)**	EXP.1	20℃/Control	18.244±0.461			86
	EXP.1	20℃/100μM Aspirin	20.674±0.613	<0.001	13.32	67
	EXP.2	20℃/Control	20.990±0.457			105
	EXP.2	20℃/100μM Aspirin	23.138±0.460	<0.001	10.23	116
	EXP.3	20℃/Control	20.548±0.548			62
	EXP.3	20℃/100μM Aspirin	24.485±0.571	<0.001	19.16	66
	**CF1903 *glp-1(e2141)III.***					
**2(C)**	EXP.1	20℃/Control	23.190±0.480			105
	EXP.1	20℃/100μM Aspirin	23.581±0.579	0.314	#	93
	EXP.2	20℃/Control	22.219±0.537			96
	EXP.2	20℃/100μM Aspirin	22.590±0.526	0.664	#	100
	EXP.3	20℃/Control	22.244±0.554			90
	EXP.3	20℃/100μM Aspirin	22.434±0.520	0.986	#	83
	EXP.4	20℃/Control	23.229±0.588			83
	EXP.4	20℃/100μM Aspirin	23.337±0.533	0.993	#	95
	**CF1038 *daf-16(mu86)I.***					
**4(C)**	EXP.1	20℃/Control	16.604±0.398			106
	EXP.1	20℃/100μM Aspirin	16.625±0.409	0.914	#	104
	EXP.2	20℃/Control	16.887±0.418			115
	EXP.2	20℃/100μM Aspirin	16.607±0.355	0.287	#	135
	EXP.3	20℃/Control	16.846±0.377			123
	EXP.3	20℃/100μM Aspirin	17.056±0.384	0.548	#	126
	**AA86 *daf-12(rh61rh411)X.***					
**4(B)**	EXP.1	20℃/Control	16.211±0.373			161

	EXP.1	20℃/100μM Aspirin	16.872±0.378	0.301	#	141
	EXP.2	20℃/Control	16.232±0.393			138
	EXP.2	20℃/100μM Aspirin	16.700±0.394	0.340	#	140
	EXP.3	20℃/Control	16.186±0.412			140
	EXP.3	20℃/100μM Aspirin	16.331±0.343	0.563	#	145
	**AA89 *daf-12(rh274)X.***					
**4(A)**	EXP.1	20℃/Control	17.903±0.581			113
	EXP.1	20℃/100μM Aspirin	17.676±0.582	0.827	#	108
	EXP.2	20℃/Control	17.820±0.550			111
	EXP.2	20℃/100μM Aspirin	16.529±0.469	0.062	#	119
	EXP.3	20℃/Control	18.400±0.543			100
	EXP.3	20℃/100μM Aspirin	17.962±0.541	0.752	#	104
	**CF1880 *glp-1(e2141)III.;daf-16(mu86)I.***					
**5(B)**	EXP.1	20℃/Control	10.900±0.323			150
	EXP.1	20℃/100μM Aspirin	11.096±0.372	0.605	#	125
	EXP.2	20℃/Control	12.083±0.361			157
	EXP.2	20℃/100μM Aspirin	11.377±0.322	0.162	#	159
	EXP.3	20℃/Control	11.966±0.368			146
	EXP.3	20℃/100μM Aspirin	11.817±0.341	0.806	#	153
	EXP.4	20℃/Control	11.392±0.362			120
	EXP.4	20℃/100μM Aspirin	11.703±0.355	0.384	#	148
	glp-1(e2141)III.; daf-12(rh61rh411)X.					
**5(A)**	EXP.1	20℃/Control	17.000±0.497			148
	EXP.1	20℃/100μM Aspirin	16.632±0.576	0.799	#	133
	EXP.2	20℃/Control	17.353±0.528			167
	EXP.2	20℃/100μM Aspirin	16.500±0.538	0.143	#	134
	EXP.3	20℃/Control	17.017±0.614			120
	EXP.3	20℃/100μM Aspirin	17.664±0.563	0.618	3.80	134
	**CF2248 *glp-1(e2141)III.; daf-12(rh61rh411)X.; daf-16(mu86)I.; muEx158.***					

**5(C)**	EXP.1	20℃/Control	11.525±0.313			181
	EXP.1	20℃/100μM Aspirin	10.920±0.302	0.182	#	175
	EXP.2	20℃/Control	11.746±0.309			197
	EXP.2	20℃/100μM Aspirin	11.874±0.315	0.928	#	183
	EXP.3	20℃/Control	11.786±0.287			220
	EXP.3	20℃/100μM Aspirin	11.270±0.296	0.310	#	200
	EXP.4	20℃/Control	11.516±0.283			217
	EXP.4	20℃/100μM Aspirin	11.479±0.325	0.799	#	188
