# Supplementary material for: Aspirin increases metabolism through germline signalling to extend the lifespan of Caenorhabditis elegans
Source: PLoS One. 2017 Sep 14;12(9):e0184027. doi: 10.1371/journal.pone.0184027 (PMC5598954; doi:10.1371/journal.pone.0184027)
Supplement: S3 Table — (PDF) [file pone.0184027.s004.pdf]

Supplementary Table 3

Figure	Strains	Treatments	Mean Lifespan ± SEM (hours)	P value VS Control	% Change in mean lifespan	N
	**N2 (WT)**					
**2(B)**	EXP.1	35℃/Control	6.926±0.377			54
	EXP.1	35℃/100μM Aspirin	9.544±0.456	<0.001	37.79	57
	EXP.2	35℃/Control	7.509±0.392			57
	EXP.2	35℃/100μM Aspirin	8.828±0.391	0.032	17.57	58
	EXP.3	35℃/Control	7.214±0.377			56
	EXP.3	35℃/100μM Aspirin	9.152±0.419	<0.001	26.86	59
	**CF1903 *glp-1(e2141)III.***					
**2(D)**	EXP.1	35℃/Control	5.717±0.144			92
	EXP.1	35℃/100μM Aspirin	5.537±0.135	0.357	#	95
	EXP.2	35℃/Control	5.853±0.135			95
	EXP.2	35℃/100μM Aspirin	5.957±0.145	0.586	#	94
	EXP.3	35℃/Control	5.692±0.136			91
	EXP.3	35℃/100μM Aspirin	5.546±0.126	0.430	#	97
	**CF1038 *daf-16(mu86)I.***					
**4(F)**	EXP.1	35℃/Control	10.379±0.370			58
	EXP.1	35℃/100μM Aspirin	10.271±0.322	0.605	#	59
	EXP.2	35℃/Control	10.370±0.359			54
	EXP.2	35℃/100μM Aspirin	10.355±0.340	0.999	#	62
	EXP.3	35℃/Control	10.107±0.358			56
	EXP.3	35℃/100μM Aspirin	10.475±0.333	0.523	3.6	59
	**AA86 *daf-12(rh61rh411)X.***					
**4(E)**	EXP.1	35℃/Control	8.517±0.401			58
	EXP.1	35℃/100μM Aspirin	8.640±0.466	0.750	#	50
	EXP.2	35℃/Control	8.222±0.415			54

	EXP.2	35℃/100μM Aspirin	8.456±0.422	0.631	#	57
	EXP.3	35℃/Control	8.194±0.349			62
	EXP.3	35℃/100μM Aspirin	8.271±0.343	0.950	#	59
	**AA89 *daf-12(rh274)X.***					
**4(D)**	EXP.1	35℃/Control	10.800±0.295			80
	EXP.1	35℃/100μM Aspirin	10.831±0.292	0.989	#	77
	EXP.2	35℃/Control	10.810±0.283			79
	EXP.2	35℃/100μM Aspirin	10.571±0.291	0.608	#	77
	EXP.3	35℃/Control	10.550±0.295			80
	EXP.3	35℃/100μM Aspirin	10.487±0.301	0.892	#	78
