# Supplementary material for: Aspirin increases metabolism through germline signalling to extend the lifespan of Caenorhabditis elegans
Source: PLoS One. 2017 Sep 14;12(9):e0184027. doi: 10.1371/journal.pone.0184027 (PMC5598954; doi:10.1371/journal.pone.0184027)
Supplement: S4 Table — (PDF) [file pone.0184027.s005.pdf]

Supplementary Table 4

Figure	Strains	Treatments	Relative intensity ±SEM	P value VS Control	N
	**N2 (WT)**				
**3(A)**	EXP.1	20℃/Control	0.982±0.007		62
**3(B)**	EXP.1	20℃/100μM Aspirin	0.909±0.008	<0.001	71
	EXP.2	20℃/Control	0.929±0.012		63
	EXP.2	20℃/100μM Aspirin	0.825±0.011	<0.001	60
	EXP.3	20℃/Control	1.089±0.012		67
	EXP.3	20℃/100μM Aspirin	0.984±0.010	<0.001	69
	**CF1903 *glp-1(e2141)III.***				
**3(C)**	EXP.1	20℃/Control	1.007±0.012		45
**3(D)**	EXP.1	20℃/100μM Aspirin	1.030±0.009	0.101	53
	EXP.2	20℃/Control	0.994±0.010		62
	EXP.2	20℃/100μM Aspirin	0.979±0.009	0.255	61
	EXP.3	20℃/Control	0.999±0.011		65
	EXP.3	20℃/100μM Aspirin	0.989±0.010	0.512	66
	**CF1038 *daf-16(mu86)I.***				
**4(I)**	EXP.1	20℃/Control	0.954±0.006		113
**4(L)**	EXP.1	20℃/100μM Aspirin	0.965±0.006	0.192	113
	EXP.2	20℃/Control	1.002±0.007		96
	EXP.2	20℃/100μM Aspirin	1.021±0.008	0.071	103
	EXP.3	20℃/Control	1.044±0.064		126
	EXP.3	20℃/100μM Aspirin	1.036±0.007	0.399	129
	**AA86 *daf-12(rh61rh411)X.***				
**4(H)**	EXP.1	20℃/Control	1.012±0.008		87
**4(K)**	EXP.1	20℃/100μM Aspirin	1.034±0.007	0.057	82
	EXP.2	20℃/Control	1.001±0.007		117
	EXP.2	20℃/100μM Aspirin	1.004±0.009	0.295	107

	EXP.3	20℃/Control	0.956±0.007		99
	EXP.3	20℃/100μM Aspirin	0.961±0.007	0.645	97
	**AA89 *daf-12(rh274)X.***				
**4(G)**	EXP.1	20℃/Control	0.969±0.008		73
**4(J)**	EXP.1	20℃/100μM Aspirin	0.987±0.009	0.144	73
	EXP.2	20℃/Control	1.052±0.008		102
	EXP.2	20℃/100μM Aspirin	1.045±0.007	0.563	106
	EXP.3	20℃/Control	0.979±0.009		79
	EXP.3	20℃/100μM Aspirin	0.973±0.009	0.657	81
