# Supplementary material for: Aspirin increases metabolism through germline signalling to extend the lifespan of Caenorhabditis elegans
Source: PLoS One. 2017 Sep 14;12(9):e0184027. doi: 10.1371/journal.pone.0184027 (PMC5598954; doi:10.1371/journal.pone.0184027)
Supplement: S5 Table — (PDF) [file pone.0184027.s006.pdf]

Supplementary Table 5

**Figure**	**Strains**	**Treatments**	**Relative intensity ±SEM**	**P value VS Control**	**N**
	**N2(WT)**				
**3(E)**	EXP.1	20℃/Control	0.957±0.037		51
**3(F)**	EXP.1	20℃/100μM Aspirin	0.782±0.026	<0.001	50
	EXP.2	20℃/Control	0.994±0.042		62
	EXP.2	20℃/100μM Aspirin	0.789±0.024	<0.001	62
	EXP.3	20℃/Control	1.049±0.047		50
	EXP.3	20℃/100μM Aspirin	0.838±0.024	<0.001	51
