# Supplementary material for: Aspirin increases metabolism through germline signalling to extend the lifespan of Caenorhabditis elegans
Source: PLoS One. 2017 Sep 14;12(9):e0184027. doi: 10.1371/journal.pone.0184027 (PMC5598954; doi:10.1371/journal.pone.0184027)
Supplement: S6 Table — (PDF) [file pone.0184027.s007.pdf]

Supplementary Table 6

Figure	Strains	Treatments	Day1	Day2	Day3	Day4	Day5	Day6	Day7	Total	N
	**N2 (WT)**										
**2(E)**	EXP.1	20℃/Control	30.83	62.07	43.50	31.36	14.50	5.68	1.67	189.61	29
	EXP.1	20℃/100μM Aspirin	31.96	62.81	45.55	30.12	16.05	4.33	1.77	192.59	28
	EXP.2	20℃/Control	17.95	77.11	68.95	47.42	17.95	3.63	0.37	233.37	26
	EXP.2	20℃/100μM Aspirin	16.74	73.68	62.26	43.42	16.21	3.74	1.05	217.11	25
	EXP.3	20℃/Control	27.52	76.13	76.04	34.43	9.17	1.83	0.74	225.87	35
	EXP.3	20℃/100μM Aspirin	27.17	73.13	73.30	38.87	10.04	0.78	0.52	223.83	37
	**Mean**	20℃/Control	25.43	71.77	62.83	37.74	13.87	3.71	0.93	216.28	
	**SEM**		3.86	4.86	9.88	4.92	2.55	1.11	0.39	13.51	
	**Mean**	20℃/100μM Aspirin	25.29	69.87	60.37	37.47	14.10	2.95	1.11	211.18	
	**SEM**		4.49	3.54	8.07	3.90	2.03	1.09	0.36	9.49	
	**P value VS Control**		0.98	0.77	0.86	0.97	0.95	0.65	0.74	0.98	
