# Supplementary material for: Aspirin increases metabolism through germline signalling to extend the lifespan of Caenorhabditis elegans
Source: PLoS One. 2017 Sep 14;12(9):e0184027. doi: 10.1371/journal.pone.0184027 (PMC5598954; doi:10.1371/journal.pone.0184027)
Supplement: S7 Table — (PDF) [file pone.0184027.s008.pdf]

Supplementary Table 7

Figure	Strains	Treatments	Mean number ± SEM	P value VS Control	N
	**N2(WT)**				
**2(F)**	EXP.1	20℃/Control	169.323±5.210		31
	EXP.1	20℃/100μM Aspirin	142.286±3.915	<0.001	35
	EXP.2	20℃/Control	157.162±3.854		37
	EXP.2	20℃/100μM Aspirin	138.750±4.412	0.002	44
	EXP.3	20℃/Control	159.844±4.811		32
	EXP.3	20℃/100μM Aspirin	142.853±4.436	0.012	34
