# Supplementary material for: Aspirin increases metabolism through germline signalling to extend the lifespan of Caenorhabditis elegans
Source: PLoS One. 2017 Sep 14;12(9):e0184027. doi: 10.1371/journal.pone.0184027 (PMC5598954; doi:10.1371/journal.pone.0184027)
Supplement: S8 Table — (PDF) [file pone.0184027.s009.pdf]

Supplementary Table 8

Figure	Strain	*sod-3*	*lipl-4*	*lips-17*	*fard-1*	*acs-2*	*ech-1.2*	*cpt-5*
	**N2(WT)**							
**3(G)**	EXP.1	1.523	2.493	1.563	1.599	5.690	2.363	1.437
**5(D)**	EXP.2	2.136	3.185	1.491	1.316	5.493	2.698	1.380
**5(E)**	EXP.3	1.849	3.033	1.874	1.367	3.813	2.431	1.299
**Mean**		1.836	2.904	1.527	1.427	4.999	2.497	1.372
**SEM**		0.177	0.209	0.117	0.151	0.421	0.072	0.028
**P value VS Control**		0.042	0.010	0.043	0.039	0.021	0.005	0.011
	**CF1903 *glp-1(e2141)III.***							
**5(D)**	EXP.1	1.068	1.236	0.807	0.988			
**5(E)**	EXP.2	1.215	1.134	1.017	1.036			
	EXP.3	1.131	1.255	1.101	1.178			
**Mean**		1.138	1.208	0.975	1.067			
**SEM**		0.043	0.037	0.087	0.057			
**P value VS Control**		0.083	0.031	0.124	0.080			
