# Supplementary material for: Aspirin increases metabolism through germline signalling to extend the lifespan of Caenorhabditis elegans
Source: PLoS One. 2017 Sep 14;12(9):e0184027. doi: 10.1371/journal.pone.0184027 (PMC5598954; doi:10.1371/journal.pone.0184027)
Supplement: S9 Table — (PDF) [file pone.0184027.s010.pdf]

Supplementary Table 9

Gene	Type	Sequence
cdc-42	F	5’-CTGCTGGACAGGAAGATTACG-3’
	R	5’-CTCGGACATTCTCGAATGAAG-3’
fat-4	F	5’-ATTGACTGGCTTTGGGGAGG-3’
	R	5’-CAGTAAGCGCATCCACCAGT-3’
hyl-1	F	5’-CCATATGCACCTCGCCTCAT-3’
	R	5’-TCGTGAATCTGAACCGTTTTCAA-3’
Y41D4A.6	F	5’-TTCCACATTTCGCGGTGACT-3’
	R	5’-GCGACGATTCTCTCCACGAT-3’
Y106GH.6	F	5’-TCGCTCCATCTCTCCCGTTA-3’
	R	5’-TTTGACGAAACCGTTGTGGC-3’
clpp-1	F	5’-GCATCTGCTGCGAAGGGTA-3’
	R	5’-GCAGAACAGGGGGCAATAGA-3’
ccr-4	F	5’-AGTTCACCAACTCGACGGAC-3’
	R	5’-AGAGAGGGAGAGCCAGTTGT-3’
egl-4	F	5’-CCGTTCCAGGCTAGTGATCC-3’
	R	5’-TTGGTGGGGTGAGAATGAGC-3’
nkb-3	F	5’-TCCAGAGGAGATCAAGGGCA-3’
	R	5’-CGAAACGAAAGAAATTGTGGGGA-3’
sod-3	F	5’-AGCATCATGCCACCTACGTGA-3’
	R	5’-CACCACCATTGAATTTCAGCG-3’
lipl-4	F	5’-ATGGCCGAGAAGTTCCTACATCGT-3’
	R	5’-GGTGAATTGGCGACCCAATCGAAA-3’
lips-17	F	5’-ATCTGTTGCTGGAGCCAATCG-3’
	R	5’-TATCCAACTTTATCGTCTCC-3’
fard-1	F	5’-GGGTTTTTGGGAAAGGTGAT-3’
	R	5’-CCACCGATTGCTTTCAATTT-3’

acs-2	F	5’-TCAATCCTCGTATCCCGCCG-3’
	R	5’-TATTCGAGGTTCCGTGGATG-3’
ech-1.2	F	5’-TGGATAAGCTCCAATCCGA-3’
	R	5’-ATTTGGATGTCAGCTCCTG-3’
cpt-5	F	5’-TCACACTGAACTGATCAAGAC-3’
	R	5’-GTAGAGCATATCGCCCTGC-3’
